# Oxidative stress and inflammation: roles in osteoporosis

**DOI:** 10.3389/fimmu.2025.1611932

**Published:** 2025-08-12

**Authors:** Jing Luo, Li Li, Wensen Shi, Kangjie Xu, Yucheng Shen, Bin Dai

**Affiliations:** Department of Central Laboratory, Binhai County People’s Hospital, Yancheng, China

**Keywords:** osteoporosis, inflammation, oxidative stress, therapeutic interventions, signaling pathways

## Abstract

Osteoporosis (OP) is a prevalent bone disease characterized by reduced bone mineral density (BMD) and compromised microstructure, leading to an increased risk of fractures and disability. With an aging global population, OP has become a significant public health issue, affecting over 200 million people worldwide. OP can be classified into primary (type I and type II) and secondary forms, with estrogen deficiency playing a critical role in postmenopausal OP. The pathophysiology of OP involves a complex interplay of factors, including cellular senescence, oxidative stress, inflammation, and hormonal imbalances. Bone homeostasis, maintained by the balance between osteoclast-mediated bone resorption and osteoblast-mediated bone formation, is regulated by various signaling pathways such as receptor activator of nuclear factor-κB ligand/receptor activator of nuclear factor-κB/osteoprotegerin (RANKL/RANK/OPG), interleukin-1/tumor necrosis factor-α (IL-1/TNF-α), and Notch. Disruption of these pathways, along with oxidative stress and chronic inflammation, leads to bone loss. Estrogen deficiency enhances pro-inflammatory cytokine production, increases osteoclast differentiation, and accelerates bone resorption. Furthermore, cellular senescence and oxidative stress contribute to reduced osteoblast function and increased adipogenesis in bone marrow mesenchymal stem cells (BMSCs). Chronic inflammation and oxidative stress further exacerbate the imbalance in bone remodeling, promoting osteoclast activity and impairing osteogenesis. Understanding the roles of immune dysregulation, oxidative stress, and inflammation in osteoporosis progression is crucial for developing targeted therapeutic strategies. This review discusses the molecular mechanisms underlying inflammation and oxidative stress in OP, highlights current therapeutic approaches, and proposes future research directions aimed at improving the prevention and treatment of osteoporosis.

## 1 Introduction

Human bone is a specialized, dynamic connective tissue characterized by high mineralization and vascularization. It continuously remodels to adapt to mechanical stress and maintain skeletal integrity. The loss of calcium and bone matrix causes osteoporosis, one of the most prevalent bone illnesses, which lowers BMD and deteriorates the microstructure (1). With global population aging, OP has become a severe health issue. The International Osteoporosis Foundation estimates that more than 200 million people worldwide have OP (2). The disease results in symptoms such as back pain, kyphosis, height reduction, and increased fracture risk. If left untreated, OP can progress silently, causing severe fractures in the wrist, hip, and spine, ultimately leading to disability and mortality. OP is also closely linked to osteoarthritis (OA), as reduced BMD and impaired trabecular mineralization can alter cartilage and subchondral bone structure. Treating OP and its complications remains a major challenge in healthcare systems worldwide.

OP is categorized as primary or secondary. Primary OP includes type I (postmenopausal) and type II (senile) forms. Type I is linked to estrogen deficiency and high bone turnover, while type II results from aging and typically involves low turnover, affecting both elderly men and women (3). Secondary OP is typically caused by underlying diseases and/or medication use, such as chronic conditions (e.g., cirrhosis, renal insufficiency, and malabsorption disorders) and drugs (e.g., anticonvulsants, heparin, thiazolidinediones, glucocorticoids, and immunosuppressants) (4, 5). Effective prevention and treatment options require an understanding of the pathophysiology of OP and how it regulates bone homeostasis.

The dynamic balance between bone resorption and production during growth, development, and repair is known as bone homeostasis, and it preserves stable bone quality and bone mineral density. Osteoblasts, which are in charge of bone creation, and osteoclasts, which are in charge of bone resorption, are the main regulators of this process. Skeletal structure and function are stable when bone production and resorption are kept in balance. This balance is regulated by multiple signaling pathways, including RANKL/RANK/OPG, mucosa-associated lymphoid tissue lymphoma translocation protein 1 (MALT1), and IL-1/TNF-α, which influence osteoclast activity (6, 7) and Notch, Hedgehog, and Wnt/β-catenin signaling pathways, which govern osteoblast development (8, 9). Disruption of these pathways leads to bone disorders such as osteoporosis.

Numerous factors, such as heredity, hormone levels, dietary condition, and lifestyle, affect bone homeostasis. Among these, sex hormones (like estrogen) are essential for preserving bone mass because they prevent bone resorption. Estrogen deficiency increases bone resorption, leading to osteoporosis. A healthy diet that includes enough calcium and vitamin D is also necessary for strong bones. The etiology of OP is complex, involving multiple factors, including estrogen deficiency, cellular senescence, oxidative stress, and chronic inflammation.

This review aims to comprehensively analyze the specific roles of inflammation and oxidative stress in the pathogenesis of osteoporosis, detailing the associated cellular and molecular mechanisms. It also summarizes current therapeutic strategies targeting inflammation and oxidative stress. Representative experimental studies elucidating the roles of inflammation and oxidative stress in osteoporosis are summarized in **Table 1**. By integrating the latest research findings, the review aims to provide a theoretical basis for precise prevention and treatment, and to offer future directions for osteoporosis research.

**Table 1:** Mechanistic studies of inflammation and oxidative stress in osteoporotic bone remodeling.

Study	Model type	Target pathways or molecules	Main findings	Reference
Effect of H_2_O_2_ on osteoblasts	*In vitro* (HEK293 cells)	mTORC1, AMPK	Low-dose H_2_O_2_ promotes proliferation via mTORC1; high-dose inhibits proliferation and suppresses Wnt/β-catenin signaling	(10)
High-fat culture environment	*In vitro* (primary murine osteoblasts)	ROS, Wnt/β-catenin	Elevated ROS inhibits Wnt/β-catenin signaling and osteoblast differentiation; promotes adipogenic gene expression	(11)
OVX mouse model	*In vivo* (ovariectomized mice)	IL-1β, TNF-α, NLRP3 inflammasome	Pro-inflammatory cytokines are upregulated; NLRP3 knockout reduces inflammatory marker expression	(12)
RANKL-induced osteoclastogenesis	*In vitro* (mouse/human osteoclast precursors)	TRAF6, NF-κB, MAPK, NFATc1	ROS enhance osteoclastogenesis through activation of NF-κB and MAPKs	(13, 14)
Ethanol-induced ROS production	*In vitro* (human osteoblasts)	RANKL/OPG	Ethanol upregulates RANKL via ROS; inhibited by NAC or 17β-estradiol	(15)
IL-6-mediated osteoclastogenesis	*In vitro* (mouse osteoclast precursors)	JAK/STAT, S1PR2	IL-6 enhances osteoclast migration and activity; upregulates S1PR2	(16)
IL-11 and bone loss	Knockout/transgenic mice	JAK1/STAT3, RANKL/OPG	IL-11 promotes bone resorption; IL-11R knockout improves bone volume and osteoblast activity	(17, 18)
IL-17-driven bone loss	*In vivo* (OVX mice)	CXCR3/CCL20, RANKL	IL-17 increases RANK sensitivity; blockade reverses OVX-induced bone loss	(19, 20)
Antioxidant NAC intervention	*In vitro* + *in vivo*	ROS, GSH/Nrf2	NAC alleviates bone erosion and oxidative stress	(21)
Drynaria fortunei extract	*In vitro* + *in vivo*	SIRT1/Notch1, NLRP3 inflammasome	Downregulates IL-1β, TNF-α; suppresses osteoclastogenesis	(22)
Resveratrol and osteogenesis	*In vitro* (human BMSCs)/animal	Wnt/β-catenin, Runx2, GSK3β	Enhances osteogenesis via β-catenin stabilization; prevents bone loss	(23)
Ebselen antioxidant therapy	*In vivo* (OA rats), *in vitro* (BMSCs, osteoclasts)	PI3K/Akt, RANKL/OPG	Inhibits osteoclastogenesis and oxidative injury; promotes osteoblast function	(24, 25)

Representative experimental studies elucidating the molecular mechanisms by which inflammation and oxidative stress contribute to osteoporosis. The table summarizes model systems, key molecular targets, primary findings, and corresponding references.

## 2 Manuscript formatting

### 2.1 Regulation of bone homeostasis by osteoblasts, osteoclasts, and key influencing factors

**Figure 1:**
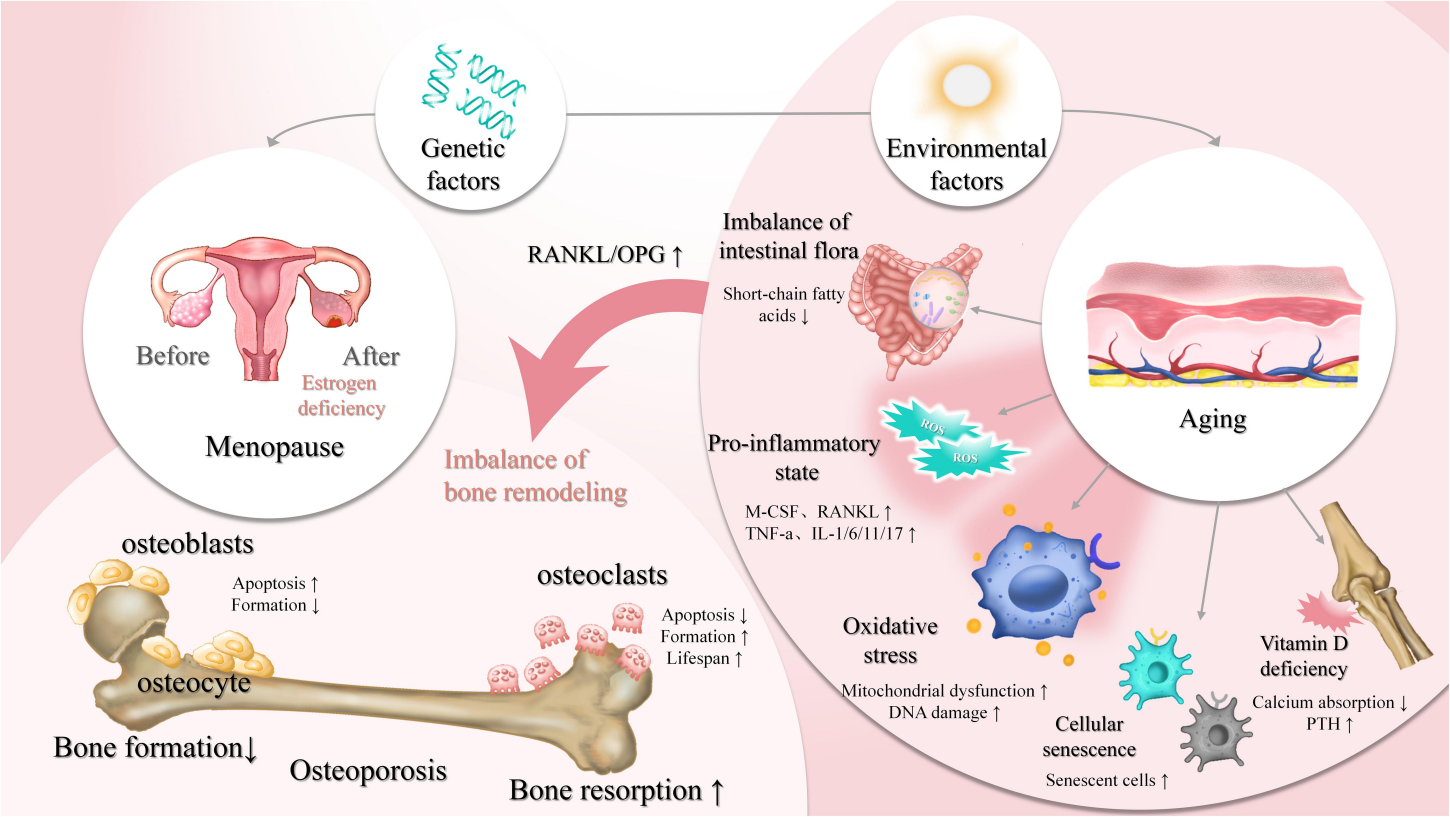
Regulation of bone homeostasis by osteoblasts, osteoclasts, and key influencing factors bone homeostasis is a dynamic equilibrium process, regulated by multiple factors such as genetics, environmental factors (e.g., aging, vitamin D deficiency), and hormonal levels (e.g., estrogen). Osteoblasts mediate bone formation, while osteoclasts are involved in bone resorption. Under normal conditions, these processes maintain bone density and mass stability. However, estrogen deficiency (e.g., post-menopause), cellular senescence, oxidative stress, and a pro-inflammatory state (e.g., elevated RANKL and TNF-α) can lead to increased bone resorption and decreased bone formation. Environmental factors like vitamin D deficiency and imbalanced intestinal flora also contribute to the disruption of bone homeostasis, thereby increasing the risk of osteoporosis. ↑, decrease; ↓, increase.

Bone homeostasis is regulated by the dynamic balance between bone-forming osteoblasts and bone-resorbing osteoclasts, and is influenced by various systemic and cellular factors (**Figure 1**). Among these, oxidative stress and inflammation have recently drawn increasing attention as central mechanisms disrupting skeletal balance and promoting osteoporosis. While classical factors such as estrogen deficiency, cellular senescence, and immune dysregulation contribute to bone loss, they often converge on or exacerbate oxidative and inflammatory pathways. Understanding how these factors interact—particularly how inflammation and oxidative stress impair osteoblast function, promote osteoclast activity, and alter the bone microenvironment—is essential for elucidating the pathogenesis of osteoporosis. The following sections examine the major regulators of bone remodeling, emphasizing the roles of oxidative stress and inflammation within this complex network.

In both men and women, estrogen insufficiency has a major role in bone loss; however, postmenopausal women are more affected because of the dramatic drop in estrogen levels (7). Serum interleukin-17A (IL-17A), RANKL, and OPG levels increase in postmenopausal women with OP, and there are also more peripheral blood CD4+ T cells that produce interleukin-17 (IL-17) (9). Estrogen exerts critical protective effects on bone mass through various mechanisms. It promotes the expression of OPG, inhibiting RANKL and preventing osteoclast formation and activity. Moreover, estrogen reduces the production of pro-inflammatory cytokines including IL-1, interleukin-6 (IL-6), and TNF-α, upregulates the bone morphogenetic protein (BMP) signaling pathway to drive mesenchymal stem cell differentiation into osteoblasts, and activates the Wnt/β-catenin pathway to promote osteogenesis. Estrogen deficiency not only impairs bone formation but also alters the expression of estrogen-target genes, leading to increased secretion of pro-inflammatory mediators that enhance bone resorption (8). As a result, estrogen is essential for sustaining bone metabolism, and its loss accelerates bone degradation.

Cellular senescence, a stable arrest of the cell cycle, limits the lifespan of proliferative cells. Hayflick and Moorhead first described this phenomenon in the 1960s, identifying telomere shortening as a natural response to prevent genomic instability and DNA damage accumulation, a process known as replicative senescence. Studies have shown that senescence markers such as p16Ink4a increase with aging in bone cells, accompanied by the accumulation of senescence-associated secretory phenotype (SASP) factors (26). As individuals age, the osteogenic differentiation capacity of BMSCs decreases, while adipogenic differentiation tends to increase. Experimental studies have demonstrated that aged mice exhibit upregulation of adipogenic genes in BMSCs, whereas osteogenic gene expression and mineralization capacity are significantly reduced. Clinical studies indicate that osteogenic potential negatively correlates with age, with younger donors displaying higher osteogenic marker expression. Aging also enhances the expression of peroxisome proliferator-activated receptor gamma 2 (PPAR-γ2), further promoting adipogenesis (27). Cellular senescence, therefore, suppresses osteogenesis and contributes to osteoporosis.

An imbalance between the production of free radicals and the antioxidant defense system—commonly observed in conditions such as postmenopausal estrogen deficiency, long-term glucocorticoid use, and aging—is known as oxidative stress. Naturally occurring byproducts of cellular oxygen metabolism and adenosine triphosphate (ATP) synthesis are reactive oxygen species (ROS). Excessive accumulation of ROS leads to oxidative stress, which adversely affects osteogenesis. In conditions like glucocorticoid therapy, osteoporosis, and other oxidative stress-related bone problems, elevated ROS levels cause osteocytes and osteoblasts to undergo apoptosis, interfere with the remodeling process, and lead to inadequate and impaired bone production. Through the activation of the extracellular signal-regulated kinase (ERK)-dependent nuclear factor-kappa B (NF-κB) signaling pathway, oxidative stress prevents osteoblast differentiation (28). To combat ROS, osteoblasts have the capacity to generate antioxidants like glutathione peroxidase. They also release transforming growth factor-beta (TGF-β), or transforming growth factor-beta, which helps to slow down bone resorption (29). As an essential component of the bone resorption process, osteoclasts, on the other hand, produce superoxide, and elevated oxidative stress promotes osteoclast activity and differentiation. Effective management of oxidative stress is crucial for maintaining bone metabolic balance (30).

Bone metabolism is closely regulated by the immunological milieu, and immune cells and the immune substances they secrete are essential for preserving the balance of bone metabolism. Under conditions of estrogen deficiency, such as in postmenopausal women, the secretion levels of RANKL by T cells and B cells are significantly increased (31). In addition to having a direct effect on bone metabolism, estrogen shortage causes pro-inflammatory cytokines like TNF-α and IL-17 to be consistently overexpressed, which results in a chronic inflammatory milieu. The expression of inflammatory factors, such as interleukin-1 beta (IL-1β), IL-6, IL-17, and TNF-α, is markedly increased as osteoporosis progresses. By increasing the expression of RANKL in osteoblasts, these pro-inflammatory substances accelerate the process of bone resorption and further enhance osteoclast differentiation and maturation (32). In ovariectomized (OVX) mice, inflammasome-related cytokines like IL-1β, interleukin-18 (IL-18), and TNF-α are markedly upregulated in bone tissues. Interestingly, in OVX mice with NLR family pyrin domain containing 3 (NLRP3) gene knockout, the expression of these pro-inflammatory factors was significantly suppressed (12). Additionally, under lipopolysaccharide (LPS) stimulation, IL-1β and IL-6 secreted by macrophages in mice were significantly increased, further contributing to the formation of a pro-inflammatory immune microenvironment (33). Investigating how immune cells and their secreted factors influence the pathogenesis of osteoporosis through the regulation of bone metabolism and the inflammatory microenvironment is essential for understanding disease progression and developing novel therapeutic strategies.

According to recent research, osteoporosis is tightly linked to oxidative stress and inflammation in addition to the conventional imbalance in bone remodeling. Inflammation and oxidative stress can upset the delicate balance between osteoblasts and osteoclasts that maintains bone metabolism, which has a major impact on both bone formation and resorption. Chronic low-grade inflammation, as observed in aging, estrogen deficiency, and autoimmune disorders such as rheumatoid arthritis, exerts a negative regulatory effect on bone cells through continuous cytokine release, while oxidative stress further exacerbates cellular damage and signaling pathway disruption via the accumulation of ROS (34). Together, immune dysfunction and inflammatory mediators drive osteoporosis progression.

### 2.2 Oxidative stress in osteoporosis

Highly reactive molecules that contain oxygen are called reactive oxygen species, or oxygen free radicals. Examples of these include hydrogen peroxide and peroxides. The regulation of apoptosis, differentiation, migration, metabolism, survival, and cell proliferation all depend on ROS. ROS act as key secondary messengers in cells, influencing various cellular functions, including regulating gene expression, controlling cell survival, and modulating intracellular signaling networks (35). While physiological levels of ROS contribute to normal cell signaling, excessive ROS—triggered by aging, inflammation, or conditions such as osteoarthritis—disrupt cellular function, promote bone loss, and ultimately lead to cell death (36).

#### 2.2.1 Oxidative stress and bone formation

Hydrogen peroxide (H_2_O_2_) inhibit osteoblast proliferation, whereas the antioxidant N-acetylcysteine (NAC) significantly restores it (37). Intervention with different concentrations of sodium fluoride in osteoblasts led to an increase in intracellular ROS levels, a decrease in proliferation, and cell cycle arrest at the S phase (DNA synthesis phase), suggesting that sodium fluoride may suppress proliferation and block cell growth by increasing oxidative stress (38). Oxidative stress speeds up bone loss by lowering osteoblast activity and quantity. A high-glucose environment temporarily enhances alkaline phosphatase (ALP) activity but reduces mineralization capacity, decreases the expression of Runt-related transcription factor 2 (Runx2), type I collagen, and osteocalcin, while upregulating adipogenic genes such as PPAR-γ (39). Furthermore, under high-fat culture conditions, ROS levels are significantly elevated, inhibiting the Wnt/β-catenin pathway and thus suppressing osteoblast differentiation (11). In differentiating mesenchymal stem cells (MSCs), the antioxidant defense system is activated to prevent excessive ROS accumulation. The system centered around Forkhead box O (FOXO) transcription factors upregulates antioxidant enzymes such as manganese superoxide dismutase and catalase and enhances autophagy to clear excess ROS (40). Studies indicate that FOXO3 mainly regulates manganese superoxide dismutase, whereas catalase expression may depend on FOXO1 or FOXO4. Additionally, autophagy alleviates ROS accumulation by clearing damaged mitochondria and toxic aggregates, thereby protecting osteoblasts. Oxidative stress, particularly in aging individuals and postmenopausal women with estrogen deficiency, is a key driver of osteoblast dysfunction, and further investigation of its specific molecular mechanisms is needed. These defense mechanisms help maintain redox balance during bone formation.

**Figure 2:**
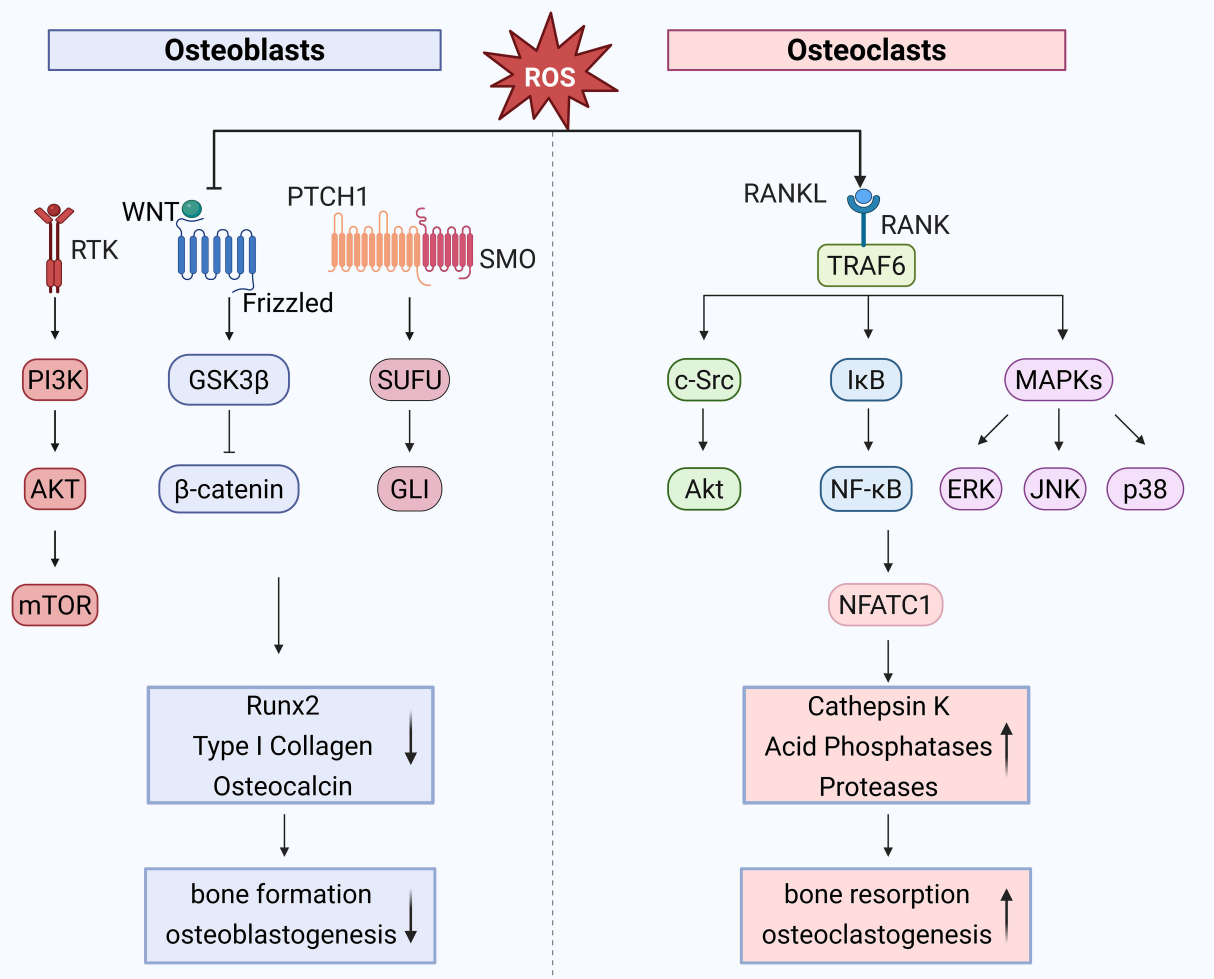
Role of oxidative stress in bone remodeling high ROS levels inhibit Wnt/β-catenin and Hedgehog signaling, impairing osteoblast differentiation. Prolonged H_2_O_2_ exposure downregulates mTOR via AMPK-mediated Raptor phosphorylation, suppressing osteoblast proliferation. In osteoclasts, ROS accumulation due to mitochondrial activity promotes differentiation via RANKL-induced activation of TRAF6, NF-κB, and MAPK pathways, enhancing NFATc1 and c-Fos expression. ROS acts as a key regulator in bone homeostasis by modulating osteoblast and osteoclast activities through interconnected signaling pathways. ↑, decrease; ↓, increase. Created with BioRender.com.

Cell proliferation is controlled by a crucial signaling protein called mammalian target of rapamycin (mTOR). Low-dose, short-term H_2_O_2_ stimulation induces an increase in ROS levels in osteoblasts, activating mTOR complex 1 (mTORC1) and promoting cell proliferation. In contrast, high-dose, long-term H_2_O_2_ stimulation significantly increases intracellular ROS levels, downregulates mTOR expression, and inhibits cell proliferation. This process is closely associated with the phosphorylation of regulatory-associated protein of mTOR (Raptor) at serine 792, mediated by AMP-activated protein kinase (AMPK) (10). Via a number of signaling pathways, oxidative stress also plays a role in controlling bone development. For instance, the Wnt/β-catenin signaling system is essential for the development and maintenance of bone, but oxidative stress reduces its activity, which impairs osteoblast differentiation (41). Furthermore, the regulation of osteoblast development under oxidative stress is significantly influenced by the Hedgehog signaling system. Oxidative stress weakens osteoblast differentiation capacity by inhibiting the Hedgehog pathway (42) (**Figure 2**). Elucidating the interplay between oxidative stress and these signaling cascades may uncover novel therapeutic targets.

#### 2.2.2 Oxidative stress and bone resorption

Osteoclasts (OCs) derive from the monocyte/macrophage progenitor cell lineage, with their differentiation process being intricate. Key factors in OC differentiation include receptor activator of nuclear factor-κB ligand (RANKL) and macrophage colony-stimulating factor (M-CSF). M-CSF is essential for the proliferation and survival of monocyte lineage cells, and it also facilitates the differentiation of OC precursors while enhancing RANK expression. RANKL binds to its receptor RANK, recruiting adapter molecules such as TNF receptor-associated factor 6 (TRAF6) and other TRAF family factors, leading to the activation of various mitogen-activated protein kinase (MAPK) signaling cascades, including ERK, p38-MAPK, and c-Jun N-terminal kinase (JNK). This activates downstream signaling, including c-Fos, which subsequently activates nuclear factor of activated T cells 1 (NFATc1), ultimately promoting the expression of various osteoclast differentiation genes (e.g., tartrate-resistant acid phosphatase (TRAP), acid phosphatase 5 (ACP5), matrix metallopeptidase 9 (MMP9), and ATPase H^+^ transporting V0 subunit d2 (ATP6)), thereby enhancing the osteoclast differentiation capacity of monocytes (43). These mechanisms emphasize the critical role of signaling networks in osteoclastogenesis and provide a framework for exploring ROS-mediated regulation.

Since osteoclasts differentiation requires a large amount of energy, OCs contain abundant mitochondria to sustain the energy demands of differentiation. ROS, primarily generated as byproducts of mitochondrial energy production, accumulate intracellularly due to reduced ROS clearance capacity caused by estrogen deficiency, aging, or other factors, thereby promoting OC activation (44). Research has demonstrated that OCs exhibit high sensitivity to ROS. During OC differentiation, RANKL increases intracellular ROS levels by activating signaling pathways involving TRAF6 and NADPH oxidase 1 (Nox1) (13). Elevated ROS levels facilitate the degradation of inhibitor of κB kinase (IκB kinase), leading to the release of NF-κB dimers and their translocation, along with NFATc1, into the nucleus. This process subsequently activates c-Fos and NFATc1, promoting OC differentiation. Additionally, ROS can activate the protein kinase B (Akt)/glycogen synthase kinase 3 beta (GSK3-β)/NFATc1 signaling cascade as well as the MAPK signaling cascade, leading to the activation of ERK, p38-MAPK, and JNK, which further stimulate downstream c-Fos and NFATc1 activation, thereby enhancing OC differentiation (14). These interconnected pathways demonstrate that ROS are not merely metabolic byproducts but active mediators of osteoclast development.

By encouraging osteoclast development and boosting osteoclast activity, ROS control bone resorption (45). Osteoporosis-induced oxidative stress is linked to decreased glutathione, decreased levels of antioxidant enzymes, and nicotinamide adenine dinucleotide phosphate (NADPH) oxidase activation, all of which promote osteoclast precursor development and bone resorption. In ovariectomized mice, oxidative stress elevates the pro-osteoclastogenic cytokine TNF-α, contributing to bone loss (46). The skeletal system’s elevated oxidative stress has also been connected to age-related bone loss in C57BL/6 mice. Moreover, studies have demonstrated that elevated ROS levels in diabetic osteoporosis enhance osteoclastic bone resorption through the activation of the MAPKs/NF-κB/NLRP3 signaling pathway (47). Therefore, increased ROS accelerate bone loss by overstimulating osteoclast function, making oxidative stress a potential therapeutic target for osteoporosis.

Osteoclast differentiation is suppressed by OPG, a soluble receptor that attaches to and blocks the receptor activator of RANKL. A growing body of research indicates that oxidative stress controls the expression of RANKL and OPG (48). Ethanol treatment increases intracellular ROS levels in osteoblasts, inducing RANKL expression and promoting osteoclast formation, while these effects can be blocked by pretreatment with 17β-estradiol or the antioxidant NAC (15). Under oxidative stress conditions, excessive ROS stimulate the expression of interleukin-6 (IL-6), which downregulates OPG expression while upregulating RANKL expression, thereby promoting bone resorption (49). The use of antioxidants such as glutathione, NAC, and lipoic acid (LA) has been shown to inhibit starvation-induced osteocyte apoptosis, downregulate RANKL expression and the RANKL/OPG ratio, and partially restore OPG expression (50). Modulating the RANKL/OPG balance may help counteract oxidative stress-related bone loss.

Hydrogen peroxide has been demonstrated to activate nuclear factor-κB, a eukaryotic transcription factor that responds to oxidative stress, in specific cell types (51). In response to external stress signals, MAPKs, a family of serine-threonine protein kinases, mediate basic biological processes and are important regulators of innate immunological and inflammatory responses. According to studies, RANKL stimulation causes osteoclasts to produce ROS, while antioxidant pretreatment lessens the activation of Akt, NF-κB, and ERK that RANKL causes (52). During osteoclast differentiation, the antioxidant arctigenin inhibits RANKL-induced MAPK activation and the activation of transcription factors such as NF-κB and NFATc1 in a dose-dependent manner, thereby suppressing the differentiation and maturation of osteoclast precursors. Moreover, iron-induced oxidative stress stimulates osteoclast development through the NF-κB pathway; this effect can be undone by inhibiting NF-κB (53). These mechanisms support the development of antioxidant-based therapies targeting ROS-related signaling pathways.

### 2.3 Inflammation in osteoporosis

**Figure 3:**
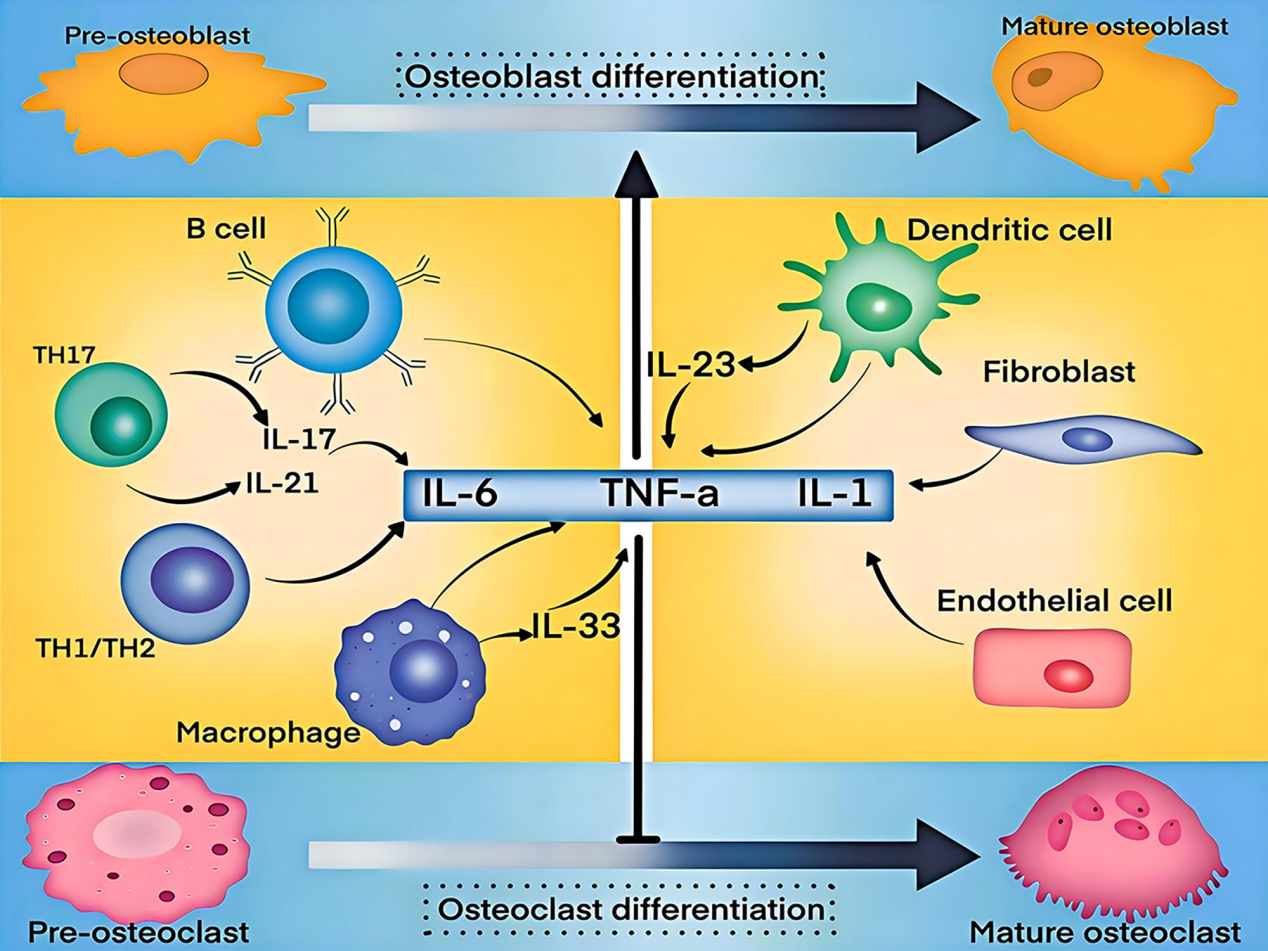
Inflammatory cytokines in bone metabolism chronic inflammation drives bone loss by promoting osteoclast activity and suppressing osteoblast function. Key cytokines, including TNF-α, IL-1, IL-6, and IL-17, disrupt bone homeostasis, contributing to osteoporosis. Emerging cytokines like IL-9, IL-21, IL-23, and IL-33 also play roles in inflammatory bone loss, warranting further investigation. Targeting these pathways may offer new therapeutic strategies for osteoporosis.

Chronic inflammation is a common cause of bone loss, and inflammation is strongly linked to bone metabolism. Reduced bone mineral density is a result of the inflammatory environment in systemic inflammatory illnesses like rheumatoid arthritis. This has been demonstrated in multiple animal models of osteoporosis, where the absence of key inflammatory mediators significantly attenuates bone loss. TNF-α, IL-1, IL-6, interleukin-11 (IL-11), and IL-17 are examples of pro-inflammatory cytokines that are essential for both immunological and inflammatory processes. Increasing evidence suggests that these cytokines influence bone metabolism through multiple mechanisms, promoting bone resorption while inhibiting bone formation, thereby serving as key drivers of osteoporosis onset and progression (**Figure 3**). Therefore, comprehending the pathophysiology of osteoporosis and creating innovative treatment approaches depend on clarifying the ways in which pro-inflammatory cytokines contribute to the condition.

#### 2.3.1 Effects of pro-inflammatory cytokines on osteoclasts

Osteoblasts produce RANKL, which controls osteoclast activation during normal bone remodeling. However, in conditions such as chronic inflammation, pro-inflammatory cytokines stimulate various cell types, such as periodontal ligament cells in periodontitis and synovial fibroblasts in arthritis, leading to increased RANKL production (54). Additionally, these cytokines can directly induce osteoclast differentiation and activation.

TNF-α, primarily produced by macrophages, promotes the inflammatory response by activating various signaling pathways within osteoclasts, including NF-κB, proteases, JNK, and MAPK, thereby inducing the release of multiple cytokines and chemokines and activating immune cells. Although TNF-α does not directly promote osteoclast development, it enhances osteoclastogenesis indirectly by upregulating RANKL expression in osteoblasts and other stromal cells (55). It also impairs osteoblast function by upregulating inhibitory proteins such as sclerostin (SOST) and Dickkopf-1 (DKK-1), which inhibit the Wnt/β-catenin signaling pathway. Conversely, blocking DKK-1 or SOST restores Wnt activity, elevates OPG levels, and reduces osteoclast differentiation (56). Thus, by altering the RANKL/OPG ratio, TNF-α enhances bone resorption, contributing to osteoporosis progression.

Interleukin-1 was initially identified as an “osteoclast activating factor” and is a highly pro-inflammatory cytokine. The majority of systemic inflammatory illnesses are caused by IL-1β, which is primarily produced by myeloid cells including mast cells, neutrophils, and macrophages in response to pro-inflammatory stimuli or autocrine stimulation (57). By upregulating the production of the chemokines C-C motif ligand 19 (CCL19), C-C motif ligand 21 (CCL21), and their receptor C-C motif chemokine receptor 7 (CCR7), IL-1 promotes osteoclast migration and activation (58). Furthermore, IL-1 promotes RANKL-dependent osteoclast development by activating transcription factors including NF-κB and activator protein-1 (AP-1) and stimulating prostaglandin E2 synthesis in osteoblasts. Additionally, *in vitro* research has verified that IL-1 directly promotes osteoclast survival, multinucleation, resorption activity, and formation (59). In TNF-transgenic mice lacking IL-1, bone damage is reduced, indicating that IL-1 is necessary for TNF-α-mediated bone loss, and IL-1 receptor antagonists can prevent this loss (60).

IL-6 is secreted by multiple bone microenvironment cells, including neutrophils, T cells, macrophages, osteoblasts, and osteoclasts. It has a close relationship with osteoclastogenesis, metastasis, and tumor invasion. IL-6 receptors exist in both membrane-bound and soluble forms, mediating local and systemic inflammatory responses (61). IL-6 binds to its receptor and the co-receptor glycoprotein 130 (gp130), directly activating Janus kinase-signal transducer and activator of transcription (JAK-STAT) and MAPK signaling pathways. This promotes the transcription of osteoclast-related proteins and the release of inflammatory cytokines, which, in turn, stimulate the NF-κB pathway, sustaining inflammation through a positive feedback loop (62). Additionally, by causing osteoblasts to produce RANKL, elevated IL-6 indirectly stimulates osteoclastogenesis. IL-6 also upregulates sphingosine-1-phosphate receptor 2 (S1PR2) expression on osteoclast precursors, facilitating their migration from the bone marrow into circulation (16). After ischemic osteonecrosis, IL-6 deficiency has been demonstrated to promote vascular regeneration and the production of new bone in mice models (63). Based on the local microenvironmental levels of RANKL, IL-6 has two roles in osteoclastogenesis: it inhibits osteoclast formation when RANKL levels are high and promotes osteoclastogenesis when RANKL levels are low (64).

Through a variety of pathways, IL-11 contributes to bone metabolism and is a crucial regulatory factor in osteolytic bone disorders (65). Bone marrow stromal cells release IL-11, which directly affects osteoclast precursors and causes osteoclastogenesis (66). Both osteoblasts and osteoclasts, as well as their precursor cells, express the full IL-11 receptor complex, consisting of interleukin-11 receptor alpha (IL-11Rα) and gp130, making them target cells for IL-11. Evidence suggests that IL-11 inhibits osteoblast-mediated bone formation and induces osteoid degradation, further triggering osteoclastogenesis (67). Additionally, by stimulating osteoclast formation via the Janus kinase 1-signal transducer and activator of transcription 3 (JAK1/STAT3) signaling pathway and c-Myc production, IL-11 stimulates bone resorption without the assistance of RANKL. It also suppresses OPG production while upregulating RANKL expression, thereby enhancing osteoclast activity (68). In pathological circumstances, illnesses like osteoarthritis and rheumatoid arthritis (RA) are intimately linked to bone loss due to aberrant IL-11 expression. The degree of bone loss is correlated with serum IL-11 levels, which are markedly higher in RA patients (69). IL-11 antagonist therapy in estrogen-deficient mice has been shown in animal studies to enhance bone quality by boosting trabecular bone volume and osteoblast activity while decreasing osteoclast quantity and function (17). Remarkably, IL-11-deficient mice do not experience the same bone loss after estrogen withdrawal, suggesting more studies are needed to clarify its precise role (18).

IL-17 is mainly secreted by T helper 17 (Th17) cells, which are elevated in the bone marrow of OVX mice with low estrogen. The growth of intestinal T lymphocytes, which go to the bone marrow via pathways involving C-X-C motif chemokine receptor 3 (CXCR3) and C-C motif ligand 20 (CCL20), is what causes this condition (20). Research indicates that IL-17 increases osteoclast precursors’ RANK expression, increasing their susceptibility to RANKL activation (70). Glutamate metabolism is also necessary for the control of IL-17, which raises energy metabolism in bone marrow-derived macrophages (BMDMs) and stimulates osteoclast development (71). It is unknown, yet, how IL-17 directly affects osteoclasts. Beyond its indirect pro-osteoclastogenic effects, Th17 cells themselves express RANKL. Blocking IL-17 signaling protects mice from OVX-induced bone loss, highlighting the essential role of IL-17 in the development and progression of postmenopausal osteoporosis. (19). Other pro-inflammatory cytokines—such as interleukin-9 (IL-9), interleukin-21 (IL-21), interleukin-23 (IL-23), and interleukin-33 (IL-33)—are also associated with inflammatory bone loss, though their mechanisms require further study (72–75).

#### 2.3.2 Effects of pro-inflammatory cytokines on osteoblasts

Through diverse molecular pathways, pro-inflammatory cytokines, including TNF-α, IL-1, IL-6, IL-11, and IL-17, control osteoblast differentiation, proliferation, apoptosis, and functional activity. These regulatory pathways involve key signaling cascades, including the Wnt/β-catenin pathway, JAK/STAT pathway, MAPK pathway, and NF-κB pathway. Clarifying how these cytokines affect osteoblast activity can deepen our understanding of bone metabolism and guide the development of targeted therapies for inflammation-related bone disorders.

TNF-α suppresses osteoblastogenesis in a number of ways. It inhibits miR-21 and semaphorin 3B (Sema3B) via Wnt/β-catenin signaling, impairing MSC osteogenic differentiation in estrogen-deficient osteoporosis, leading to reduced bone mineral density and trabecular abnormalities (76). Additionally, TNF-α directly inhibits osteoblast differentiation by downregulating osteogenic transcription factors such as special AT-rich sequence binding protein 2 (SATB2), Runx2, and osterix (Osx). Furthermore, TNF-α suppresses osteogenesis by upregulating Smad ubiquitination regulatory factor 1 (Smurf1) and Smurf2, which cause the RUNX2 protein to be ubiquitinated and degraded by proteasomes (77). Interleukin-1 alpha (IL-1α) inhibits osteoblastogenesis and promotes cell death by activating JNK and p38/MAPK pathways (78). IL-1β inhibits osteoblast recruitment and suppresses osteoblast differentiation via the MAPK and NF-κB pathways while promoting tumor-induced bone metastasis. IL-6 is a multifunctional cytokine with complex effects on osteoblasts. IL-6 negatively regulates osteoblast differentiation *in vitro* through the SHP2 (Src Homology 2 domain-containing phosphatase 2)/mitogen-activated protein kinase 2 (SHP2/MEK2) and SHP2/Akt2 pathways (79). IL-6 released by osteosarcoma cells also prevents BMSCs from undergoing osteogenic differentiation (80). However, previous studies suggested that IL-6-type cytokines promote the differentiation of committed osteoblasts into more mature phenotypes, primarily through JAK/STAT pathway activation (81). IL-6 also suppresses osteoblast gene expression—such as ALP, Runx2, and osteocalcin—through PI3K/Akt2 and MEK/ERK signaling, reducing new bone mineralization. IL-11 promotes osteoblast lineage commitment while suppressing adipogenesis, and regulates osteoblasts under mechanical stress via Wnt/β-catenin signaling (82). IL-11 receptor knockout mice exhibit shortened long bone length and reduced overall body size. Conversely, transgenic mice overexpressing human IL-11 show increased bone formation, enhanced cortical mass and thickness, and improved long bone strength (83). IL-17 also plays a role in osteoclastogenesis with the assistance of osteocytes. However, as demonstrated by increased extracellular matrix calcium deposition and alkaline phosphatase activity, IL-17 also has osteogenic effects, encouraging osteoblast development and maturation (84). IL-17A stimulates osteoblast development through the JAK2/STAT3 pathway in ankylosing spondylitis (85). Therapeutic strategies targeting these cytokines may help mitigate inflammation-related bone diseases.

### 2.4 Interventional strategies for inflammation and oxidative stress

#### 2.4.1 Anti-inflammatory drugs

Inflammatory diseases such as ankylosing spondylitis and rheumatoid arthritis commonly cause bone loss through complex immune and inflammatory signaling pathways. Although conventional anti-osteoporotic drugs like bisphosphonates and calcitonin can reduce bone loss, they do not address the underlying inflammation. Nonsteroidal anti-inflammatory drugs (NSAIDs), as first-line treatments for inflammatory diseases, exert anti-inflammatory and analgesic effects primarily by inhibiting cyclooxygenase (COX) and reducing prostaglandin synthesis (86). However, NSAIDs alone often fail to sufficiently alleviate symptoms or improve patients’ quality of life, especially in cases of persistent inflammatory activity. Biologic disease-modifying anti-rheumatic drugs (DMARDs), including TNF-α and IL-6 inhibitors, have become critical for targeting specific pro-inflammatory cytokines, effectively suppressing the inflammatory cascade and reducing bone loss. Glucocorticoids, widely used as classical anti-inflammatory agents, exhibit potent anti-inflammatory and analgesic properties. However, high-dose or long-term use can lead to osteoporosis by increasing the expression of M-CSF and RANKL while inhibiting osteoblast-related signaling pathways such as Wnt/β-catenin. Recent advances in immunotherapy provide novel strategies for preventing inflammation-induced bone loss. By modulating pro-inflammatory cytokines and their downstream signaling pathways, immunotherapeutic approaches can suppress osteoclast activation and restore the balance of bone remodeling, offering a promising strategy for treating chronic inflammation-related osteoporosis. Understanding the mechanisms underlying inflammation-targeted therapies, particularly those focusing on inflammatory cells and pro-inflammatory factors, is crucial for elucidating the pathological basis of inflammatory bone loss and developing novel therapeutic interventions.

Since TNF-α inhibitors can lessen inflammation and prevent systemic bone loss, they are frequently used in clinical practice as the first-line therapy for rheumatoid arthritis. Investigations have revealed that the TNF-α blocker infliximab has a certain effect on bone metabolism; however, its impact on bone biomarkers remains inconsistent. Some studies indicate that short-term infliximab treatment (14 weeks) decreases both bone formation markers, such as osteocalcin, and bone resorption markers, such as N-terminal telopeptide (NTX) (87). However, additional research shows that infliximab inhibits bone resorption markers such as C-terminal cross-linked telopeptide of type I collagen (ICTP), while increasing bone formation markers including osteocalcin and procollagen type 1 N-terminal propeptide (P1NP). No appreciable changes in BMD at the hip or lumbar spine have been noted, despite the fact that a year of infliximab therapy causes a decrease in bone resorption indicators (C-terminal cross-linked telopeptide of type I collagen, CTX-1) and an increase in bone formation markers (88). These conflicting results suggest substantial heterogeneity in the biological response to infliximab, which may be attributed to variations in study design, treatment duration, and especially patient-specific factors such as menopausal status, baseline disease activity, and concurrent medication use. Moreover, some studies lack standardized protocols for biomarker assessment, making cross-study comparisons difficult. Beyond its role in RA, infliximab has demonstrated potential in preventing tumor-induced bone metastasis by reducing the viability of metastatic breast cancer cells (e.g., MDA-MB-231) and effectively inhibiting breast cancer bone metastasis in mouse models (89). TNF-α inhibitors not only improve inflammation-associated bone metabolic disorders but also show therapeutic potential in inflammatory bone loss diseases such as RA, inflammatory bowel disease (IBD), and ankylosing spondylitis (AS) (90, 91). Commonly used TNF-α inhibitors include adalimumab, which specifically binds to TNF-α, and etanercept, which competitively inhibits TNF receptor (TNFR) signaling (92). Despite their broad use, the clinical efficacy of TNF-α inhibitors in preventing structural bone damage remains a matter of debate. For example, while some studies report improved bone turnover profiles, others fail to demonstrate significant gains in BMD or fracture risk reduction, especially in long-term follow-up (93). While TNF-α inhibitors are generally well tolerated, they may lead to severe adverse effects such as infections and malignancies (94). This raises important safety concerns, particularly in elderly or immunocompromised populations. Furthermore, the long-term effects on skeletal integrity remain unclear, with insufficient real-world data to guide personalized risk-benefit assessments. To enhance therapeutic efficacy and minimize risks, future research should explore selective targeting of TNFR1 or activation of the TNF/TNFR2 pathway, which may provide a safer and more effective means of suppressing inflammation (95). Additionally, optimizing treatment regimens is essential to reducing side effects and improving long-term patient outcomes, better meeting clinical needs.

IL-1 inhibitors effectively block IL-1 signaling, thereby reducing cartilage and bone destruction. Anakinra is a recombinant IL-1 receptor antagonist that inhibits the activity of IL-1α and IL-1β. It is commonly used, either alone or in combination with other medications, to treat conditions such as neonatal-onset multisystem inflammatory disease, rheumatoid arthritis, and other inflammatory disorders like cryopyrin-associated periodic syndromes (CAPS). While it has been shown to reduce systemic bone loss, its impact on bone metabolism is generally less pronounced compared to other biologics targeting cytokines. Importantly, most studies evaluating the bone-protective effects of IL-1 inhibitors are based on small cohorts or preclinical models, which limits the generalizability of their findings. Additionally, the modest efficacy observed in RA-related bone preservation has raised questions regarding whether IL-1 inhibition alone is sufficient to halt structural joint damage or restore bone microarchitecture in advanced disease stages (96). Soluble receptor blockers such as rilonacept and anti-IL-1β monoclonal antibody canakinumab also exert anti-inflammatory effects by neutralizing IL-1 (97). A bioinformatics study on AS-associated osteoporosis suggests that rilonacept may target genes related to both AS and osteoporosis, indicating its potential for preventing or treating bone loss in AS patients (98). Additionally, canakinumab reduces tumor cell migration to bone by blocking IL-1β from binding to its receptor, significantly decreasing the incidence and progression of breast cancer bone metastases (99). Although promising, these anti-metastatic effects have so far been observed predominantly in preclinical settings, and no large-scale clinical trials have confirmed the protective role of canakinumab in human skeletal metastasis. Emerging research indicates that IL-1 receptor antagonist (IL-1Rα) may play a direct role in osteoblast differentiation in osteoporosis. IL-1Rα not only competitively inhibits IL-1 binding to its receptor, thereby disrupting IL-1-induced osteoclastogenesis, but also directly interacts with integrin β3, activating downstream β-catenin signaling and inducing the expression of osteogenic markers (100). These mechanistic insights open new avenues for repositioning IL-1 antagonists as osteoanabolic agents; however, the physiological relevance of these pathways under chronic inflammatory conditions and in the aging skeleton remains uncertain. Further experimental validation in animal models of osteoporosis and prospective clinical studies are essential to determine whether such effects translate into meaningful improvements in bone mass or fracture risk.

IL-6 inhibitors play a crucial role in the treatment of rheumatoid arthritis by reducing inflammation-induced osteoporosis through the suppression of osteoclast activity. Tocilizumab, an IL-6 receptor (IL-6R) antagonist, has been shown to stimulate the bone formation marker P1NP while inhibiting bone resorption markers CTX-1 and ICTP after four weeks of treatment (101). A long-term study demonstrated that two years of tocilizumab treatment increased femoral neck BMD but had no significant effect on lumbar spine BMD while reducing CTX levels, with no observed changes in osteocalcin or P1NP levels (102). Additionally, lumbar spine and femoral neck BMD significantly increased in patients with osteopenia, whereas no significant changes were observed in those without osteopenia (103). These findings highlight a potentially differential benefit of IL-6R blockade on bone health depending on baseline bone status, suggesting that patients with pre-existing low bone mass may experience more substantial gains. However, the lack of consistent improvement in bone turnover markers (such as osteocalcin and P1NP) across studies points to variability in individual responses, potentially due to age, sex, disease duration, glucocorticoid co-treatment, or hormonal status. Sarilumab targets both soluble IL-6R (sIL-6R) and membrane-bound IL-6R (mIL-6R), making it the second IL-6R antagonist authorized after tocilizumab. Either alone or in conjunction with traditional synthetic disease-modifying antirheumatic medications (csDMARDs), it can be utilized (104). Sarilumab has been widely applied in RA treatment, demonstrating significant benefits in improving joint mobility (as measured by ACR20, ACR50, and ACR70 response rates, indicating 20%, 50%, and 70% improvements, respectively, based on the American College of Rheumatology (ACR) criteria.) and slowing disease progression (as assessed by the modified Sharp/van der Heijde score) (105). By blocking IL-6 signaling, sarilumab significantly increases bone formation markers, including P1NP and osteocalcin, while reducing total RANKL levels in the early stages of treatment, with sustained suppression lasting up to 24 weeks. This leads to a marked decrease in bone resorption markers such as CTX-I and ICTP (106). Furthermore, in RA patients, sarilumab improves the overall balance of bone biomarkers, significantly reducing CTX-I and stabilizing or slightly increasing BMD in some cases (107). In collagen-induced arthritis (CIA) animal models, IL-6R blockade has been shown to reduce the number of osteoclast precursors in the bone marrow, prevent bone loss, and preserve bone strength (108). While these preclinical findings provide valuable mechanistic insights, extrapolation to human disease must be approached with caution. In particular, differences in immune environment and bone remodeling dynamics between rodents and humans may influence treatment outcomes. Furthermore, it remains unclear whether IL-6 inhibitors can promote net bone formation or simply suppress inflammation-mediated resorption. Long-term use of IL-6 inhibitors provides significant benefits in improving bone density and reducing bone resorption markers, offering a promising therapeutic option for RA patients.

A completely human IgG1 monoclonal antibody called secukinumab binds to and neutralizes IL-17A specifically. It is authorized to treat psoriatic arthritis (PsA) and moderate to severe AS. In a phase III trial for AS, secukinumab significantly improved ASAS20 response rates, reaching 60% at week 16 compared to 37% in the placebo group, with sustained efficacy up to week 52 (109). In PsA studies, secukinumab significantly slowed radiographic disease progression within 24 weeks and inhibited joint destruction over 52 weeks compared to placebo (110). While these structural benefits suggest a protective effect on bone and cartilage, direct evidence evaluating secukinumab’s impact on BMD, bone turnover markers, or fracture risk is currently lacking. Most conclusions are extrapolated from imaging outcomes such as joint space narrowing or erosion scores, which, although important, do not necessarily equate to improved systemic bone health. Psoriasis, PsA, and AS have all been treated with IL-17A monoclonal antibodies (like secukinumab and ixekizumab) and IL-17 receptor A monoclonal antibodies (like brodalumab), which have shown promise in reducing inflammation-induced bone loss. However, the bone-protective effects of these agents remain largely inferential and are not yet supported by robust longitudinal data on BMD or fracture outcomes in human populations. Moreover, the relative contribution of IL-17 inhibition to osteoclast suppression versus potential osteoblast stimulation remains poorly defined. These IL-17 inhibitors provide a different kind of therapy for people who don’t react to TNF-α inhibitors. However, mild to moderate infections are common adverse effects, and some patients may experience severe infections (111). While IL-17A monoclonal antibodies show promise in improving inflammatory osteoporosis, their long-term efficacy and safety require further investigation to fully establish their clinical value and optimize treatment strategies.

One important cytokine-regulated signaling cascade that is essential for triggering innate immunity, coordinating adaptive immune processes, and eventually reducing inflammation and immunological responses is the JAK/STAT pathway (112). Broad-spectrum JAK inhibitors, such as tofacitinib, peficitinib, ruxolitinib, and baricitinib, have shown promising therapeutic potential for inflammatory, immune, and hematologic disorders (113–117). By inhibiting the JAK/STAT pathway, these agents simultaneously block multiple cytokine-driven inflammatory pathways, reducing the transcription of pro-inflammatory cytokines, thereby effectively alleviating inflammation while potentially mitigating adverse effects (118). It is generally known that tofacitinib is safe and effective in treating moderate to severe active rheumatoid arthritis (119). However, despite its anti-inflammatory benefits, there is a notable lack of clinical studies specifically evaluating its effects on local or systemic bone mass, BMD, or fracture outcomes. This represents a major gap in understanding whether its immunomodulatory actions translate into meaningful skeletal protection. An assessment of tofacitinib in a rat model of adjuvant-induced arthritis revealed that although it reduced inflammation, it was unable to stop the loss of cortical and trabecular bone strength and structure brought on by inflammation (120). This dissociation between anti-inflammatory efficacy and bone-preserving capacity suggests that suppression of systemic inflammation may not be sufficient to reverse or prevent skeletal damage, especially in models with advanced joint destruction. In contrast, peficitinib demonstrated significant bone-protective effects in the same model, markedly reducing bone destruction scores and nearly completely preventing paw swelling and bone damage at a dose of 30 mg/kg (121). In adjuvant-induced arthritic rat models, baricitinib has been demonstrated to restore joint structure and stop bone loss by blocking intracellular signaling of many pro-inflammatory cytokines, such as IL-6 and IL-23 (122).

The NF-κB signaling pathway is a vital mechanism that cells use to regulate responses to inflammation, immune reactions, oxidative stress, as well as processes like cell proliferation, differentiation, and apoptosis. This pathway is integral to maintaining cellular homeostasis and is involved in various physiological functions. Its dysregulation can lead to chronic inflammation and disease progression (123). The IκBα phosphorylation and NF-κB inhibitor BAY 11-7082 (also called BAY 11-7821) selectively and irreversibly inhibits TNF-α-induced IκBα phosphorylation, which lowers the expression of adhesion molecules and NF-κB. Research findings suggest that silencing long non-coding RNA CRNDE exacerbates IL-1β-induced chondrocyte damage, whereas the NF-κB inhibitor BAY 11-7082 can mitigate this damage. Furthermore, by blocking the NF-κB signaling pathway, the NF-κB inhibitor JSH-23 inhibits RANKL-induced osteoclastogenesis and bone resorption. Interestingly, JSH-23 also inhibits ROS generation, reducing H_2_O_2_-induced osteoblast apoptosis and mineralization loss (124). Additionally, by blocking the NF-κB signaling pathway, paroxetine therapy inhibits the pyroptosis-related pathway triggered by IL-1β and reduces the extracellular matrix disintegration. Osteoclast production is thus decreased, as is the expression of osteoclast marker genes triggered by RANKL (125). However, most current data derive from *in vitro* or small animal models, which may not accurately replicate the complexity of human bone remodeling or chronic inflammatory microenvironments. Moreover, the specificity of small-molecule inhibitors like BAY 11-7082 and JSH-23 is often questioned, as off-target effects and cytotoxicity may confound interpretation of results (126).

#### 2.4.2 Antioxidant drugs

By directly interacting with free radicals or indirectly by preventing the activity of enzymes that produce free radicals or by increasing the activity of intracellular antioxidant enzymes, antioxidants can mitigate the symptoms of osteoporosis. These steps aid in lowering oxidative stress, which is a major cause of bone loss. Antioxidants can be broadly classified into two categories: naturally sourced and synthetically produced. Naturally sourced antioxidants are primarily derived from medicinal plants and phytochemicals found in dietary sources, which possess antioxidant properties. Synthetic antioxidants are laboratory-designed to improve bioavailability, stability, and bone-targeting efficiency.

##### 2.4.2.1 Natural antioxidants

Natural antioxidants inhibit bone resorption, support bone remodeling, and promote bone formation. They are believed to enhance matrix synthesis, stimulate osteoblast differentiation, and suppress osteoclast activity, offering potential for osteoporosis prevention and treatment (127).

Inhibiting oxidation and microbiological development are two important functions of natural antioxidants. They are composed of a variety of substances, including vitamins, natural colors, and polyphenols. Lipoic acid, uric acid, glutathione, and certain amino acids are examples of endogenous antioxidants that support cellular redox equilibrium and provide protection against oxidative stress in addition to the previously listed exogenous antioxidants. In addition to having a substantial positive impact on general health, these natural substances may have therapeutic value in preventing or treating diseases like osteoporosis that are linked to oxidative stress.

Polyphenols-classified divided into flavonoid and non-flavonoid groups are abundant in fruits and plants. The anti-inflammatory and antioxidant properties explain their bone-protective roles (128). Oxidative stress occurs when the production of ROS exceeds the capacity of antioxidant defense systems. With their many phenolic hydroxyl groups, polyphenolic substances function as hydrogen donors, converting singlet oxygen into the less active triplet oxygen form. This lowers the probability of producing free radicals and stops chain reactions brought on by them (129). According to research, consuming berries that are naturally high in dietary polyphenolic components, including blueberries and cranberries, can help prevent and cure osteoporosis by scavenging free radicals and reducing oxidative stress (130). It has been demonstrated that plum and its polyphenolic chemicals directly decrease osteoblastogenesis by downregulating NFATc1 and inflammatory mediators, which lowers osteoblast activity, and limit bone resorption by downregulating RANKL (131). Icariin and resveratrol, extensively studied for their bone-protective effects, regulate BMSC osteogenic differentiation, prevent bone loss, and promote bone regeneration (132). Icariin activates autophagy and exerts anti-inflammatory effects, alleviating osteoporosis. Furthermore, icariin intervention in rat BMSC significantly upregulates osteogenic genes and activates the Wnt signaling pathway. Resveratrol maintains the stability of β-catenin in the cytoplasm, which translocates to the nucleus to activate the Wnt/β-catenin pathway, by upregulating Runx2 expression, downregulating GSK3β, and preventing the efficient assembly of β-catenin degradation complexes. This aids in the prevention and treatment of osteoporosis by encouraging osteoblast development and increasing bone mineral density (23). Punicalagin, from pomegranate, shows antioxidant and anti-inflammatory effects by reducing TNF-α-induced ROS, promoting osteogenesis in BMSCs, and inducing angiogenesis in Human Umbilical Vein Endothelial Cells (HUVECs). It also modulates immune responses via Nrf2/HO-1 (Nuclear factor erythroid 2-related factor 2/Heme oxygenase-1) signaling, promoting M2 macrophage polarization (133). Apples and strawberries are among the fruits that naturally contain rutin, a phenolic component. ALP, osteocalcin, type I collagen, Runx2, and osterix are examples of osteoblast differentiation indicators whose mineralization and expression are inhibited by rutin treatment, whereas adipogenic markers are increased (134). Procyanidin, which is present in grape seeds, suppresses NF-κB and JNK signaling, which prevents osteoclast development and the differentiation of RAW264.7 cells and bone marrow macrophages (135). Procyanidin-rich grape seed extract (GSE), when taken orally once a day, improves implant osseointegration in OVX rats, encourages bone repair, and stops vertebral and femoral bone loss in OVX mice (136). Despite the abundant preclinical evidence supporting the bone-protective effects of various polyphenols, translation to clinical practice remains limited by several factors. Clinical trials investigating polyphenol supplementation in human osteoporosis are sparse and often yield inconsistent results, partly due to differences in dosage, bioavailability, and formulation. Moreover, many studies rely on *in vitro* or animal models, which may not fully replicate human bone metabolism or disease complexity. The dual role of some polyphenols, such as rutin’s inhibition of osteoblast differentiation and simultaneous promotion of adipogenesis, further underscores the complexity and potential unintended effects of these compounds. Furthermore, the mechanisms by which these polyphenols modulate bone remodeling involve intricate signaling pathways, including Wnt/β-catenin, NF-κB, and Nrf2/HO-1, which also participate in systemic physiological processes. This raises concerns about off-target effects and necessitates a careful assessment of long-term safety and interaction with conventional osteoporosis medications.The prevention and treatment of osteoporosis may benefit greatly from the use of polyphenolic compounds because of their remarkable anti-inflammatory and antioxidant qualities. More scientific proof and treatment approaches for the complete prevention and management of osteoporosis will come from future studies that examine the mechanisms and clinical uses of different polyphenols.

Lycopene is a fat-soluble carotenoid primarily found in tomatoes. It has been shown to reduce osteoclast differentiation without affecting cell viability, and it can enhance osteoblast proliferation and differentiation by modulating the MEK signaling pathway in both osteoblasts and osteoclasts (137). Lycopene is one of the most powerful antioxidants among carotenoids, as it activates the WNT/β-catenin and ERK1/2 pathways, upregulating RUNX2, ALP, and type I collagen, while downregulating RANKL. Consumption of lycopene-rich tomato sauce significantly prevented bone loss in a study involving 39 postmenopausal women (138). A 4-month clinical research involving 60 postmenopausal women also showed that lycopene capsule users had a considerably decreased incidence of osteoporosis. Lycopene may present a potential strategy for osteoporosis prevention and therapy, according to these data (139).

Bone homeostasis is maintained by vitamin D, especially VD2 and VD3, which are fat-soluble steroids essential for bone health (140). The vitamin D receptor (VDR), a nuclear receptor that controls target gene transcription, is the main mechanism by which they have biological effects (141). VDR is extensively dispersed throughout the body’s tissues and cells, and it contributes significantly to bone homeostasis by controlling osteoblasts and osteoclasts. VDR activation in osteoblasts promotes the synthesis of extracellular matrix proteins, such as type I collagen, osteopontin, and osteocalcin, and increases the expression of osteogenic markers including Runx2, Osx, and ALP (142). VDR activation also leads to a decrease in RANKL expression and an increase in OPG expression, thereby inhibiting osteoclastogenesis and maintaining bone homeostasis (143). In addition to lowering the expression of genes linked to osteoclast activity, VDR activation in osteoclasts lowers the synthesis of RANKL and M-CSF, preventing osteoclast development and subsequent bone resorption (144). The equilibrium between bone production and resorption is therefore preserved by VDR activation. Vitamin E is an antioxidant fat-soluble vitamin that comes in tocopherols (α-, β-, γ-, and δ-) and tocotrienols (α-, β-, γ-, and δ-). BMD and circulating α-tocopherol levels have been found to be significantly positively correlated by Mendelian randomization experiments (145). Considering that vitamin E is regarded as a risk factor for osteoporosis in postmenopausal women, a cross-sectional investigation revealed that low blood vitamin E levels were linked to lower BMD. α-Tocopherol supplementation markedly promotes fracture healing in OVX mice. (146). Additionally, in a rabbit distraction osteogenesis model, supplementation with α-tocopherol significantly increased bone density, osteogenesis, and osteoblast activity (147). Despite these promising findings, the dual role of vitamin E—particularly α-tocopherol—in bone metabolism has raised concern. Some animal and *in vitro* studies have reported pro-oxidant effects or suppression of osteoblastogenesis at higher doses. Moreover, discrepancies between tocopherols and tocotrienols in their bioactivity and mechanisms remain underexplored. Therefore, dose optimization and comparative evaluation of different isoforms are essential before recommending vitamin E supplementation for osteoporosis management (148). The fat-soluble vitamin K contains menaquinone (VK2) and phylloquinone (VK1). Plants are the primary source of VK1, whereas fermented foods are the main source of VK2. Additionally, there are 12 MK subtypes of VK2, with MK-4 and MK-7 shown to promote the proliferation of osteoblast precursors, enhance alkaline phosphatase activity, stimulate osteocalcin synthesis, and increase calcium deposition, thereby supporting bone health (149). Studies have shown that by lowering blood levels of uncarboxylated osteocalcin (uc-OC), VK2 considerably increases the BMD of the lumbar spine. VK2 (MK4 or MK7) causes osteocalcin to undergo γ-carboxylation, resulting in carboxylated osteocalcin (cOC), which may strengthen bones by encouraging mineralization (150). Supplementation with vitamin K2 appears beneficial and safe for treating osteoporosis in postmenopausal women. However, current clinical evidence supporting vitamin K2’s efficacy is still evolving. While some trials have shown improvements in BMD and reductions in uc-OC, robust data on fracture risk reduction are limited. Additionally, the interplay between vitamin K and anticoagulant therapy, especially vitamin K antagonists, must be carefully considered in elderly patients who are at high risk for both osteoporosis and cardiovascular disease. Vitamin C, through *in vitro* and animal models, has been shown to influence osteoporosis. It functions by inhibiting osteoclast activity, stimulating type I collagen synthesis, promoting osteoblast maturation, and regulating gene transcription, DNA, and histone methylation (151). Vitamin C supplementation after ovariectomy in mice prevents bone loss and osteoblast depletion (152). The osteogenic differentiation regulator Osx is also elevated in bone marrow stromal cells following vitamin C consumption, which causes Nrf1 to bind to the antioxidant response element (ARE) in the Osteix promoter (153). Nevertheless, clinical data on the role of vitamin C in osteoporosis prevention or treatment remain sparse. Observational and interventional studies yield inconsistent results, partly due to confounding factors such as dietary patterns, smoking status, and baseline vitamin C levels. Furthermore, the bioavailability of high-dose oral vitamin C and its actual impact on bone mineral density or fracture outcomes in human populations remain unclear. Additional large-scale, controlled trials are needed to clarify its potential therapeutic value.

##### 2.4.2.2 Synthetic antioxidants

Synthetic antioxidants are substances that are created in labs and are usually intended to improve particular therapeutic qualities like increasing bioavailability, lengthening the duration of action, or facilitating targeted administration. Compared to natural antioxidants, synthetic antioxidants offer more controllable properties and higher stability. They have shown great promise in the prevention and treatment of osteoporosis, especially in boosting bone formation, lowering inflammation linked to oxidative stress, and blocking bone resorption. These compounds are being explored for their ability to not only mitigate oxidative damage but also to modulate bone metabolism and offer effective therapeutic strategies for managing osteoporosis and related bone disorders.

Ebselen is a non-toxic selenium-based organic drug and an enzyme mimic, initially developed to simulate the action of glutathione peroxidase (GPx) in reducing peroxides. It exhibits both anti-inflammatory and antioxidant properties. Studies have shown that ebselen inhibits osteoclastogenesis by regulating the RANKL/OPG ratio. Additionally, ebselen induces osteoclast apoptosis in the later stages of osteoclast differentiation (24). Ebselen also alleviates H_2_O_2_-induced osteogenic dysfunction in BMSCs through its antioxidant effects and by activating the PI3K/Akt pathway (25). Ebselen treatment has been shown to reduce joint cartilage degeneration in a rat knee OA model (154). However, the majority of available data come from preclinical studies, and human clinical evidence supporting ebselen’s bone-protective effects remains limited. Moreover, the long-term impact of selenium accumulation in the context of chronic administration is still not fully understood. Edaravone reacts with peroxides and hydroxyl radicals to produce oxidized molecules and is a powerful free radical scavenger. In the supernatant of monocyte and synovial fibroblast co-cultures, edaravone decreases the amounts of cytokines, osteopontin (OPN), RANKL, and M-CSF and suppresses osteoclast development (155). *In vivo*, edaravone treatment promotes H_2_O_2_-induced cell proliferation and enhances osteoblast differentiation (156). Nonetheless, edaravone’s pleiotropic effects may complicate its therapeutic targeting, and data regarding its pharmacokinetics and optimal dosing in bone disease remain scarce. NAC, a precursor for glutathione (GSH) synthesis, is a small molecular amino acid derivative. NAC enhances osteogenic differentiation, upregulates GSH levels, and increases the GSH/GSSG (Glutathione disulfide, GSSG) ratio. It also downregulates ROS generation and Nrf2 expression induced by cyclic mechanical stress in periodontal ligament stem cells (PDLSCs) (21). In a LPS-induced inflammatory bone resorption mouse model, NAC treatment alleviates bone erosion and protects mice from LPS-induced bone resorption. Although synthetic antioxidants show great potential in osteoporosis treatment, their use faces challenges. For example, the long-term safety, potential toxicity, and biodegradability of these compounds require further evaluation. Furthermore, the development costs of synthetic antioxidants are greater than those of natural antioxidants, and additional randomized controlled trial (RCT) evidence is still needed to justify their usage in clinical settings.

### 2.5 Inflammation- and oxidative stress–associated biomarkers and therapeutic strategies in osteoporosis

Recent clinical studies have identified several circulating biomarkers and imaging indicators associated with oxidative stress– and inflammation-mediated bone loss. These biomarkers not only deepen our understanding of the underlying pathophysiological mechanisms but also hold promise as tools for treatment monitoring and patient stratification. Key oxidative stress– and inflammation-related biomarkers, along with their corresponding therapeutic interventions evaluated in clinical studies, are summarized in **Table 2**.

**Table 2:** Representative clinical biomarkers and intervention strategies related to inflammation and oxidative stress in osteoporosis.

Biomarker/pathway	Type	Role in bone metabolism	Intervention strategy	Clinical stage	Reference
MDA (Malondialdehyde)	Oxidative stress marker	Lipid peroxidation product; elevated in high ROS states, linked to bone loss	Melatonin 20 mg/day for 12 months	RCT completed	(157)
8-OHdG	Oxidative DNA damage marker	Indicates oxidative DNA injury; correlated with bone resorption	Tocotrienol supplementation (300–600 mg/d)	Phase II RCT (NCT02058420)	(158)
sRANKL/OPG ratio	Osteoclast regulatory ratio	High ratio indicates enhanced osteoclast activation	Tocotrienol reduced the ratio in postmenopausal women	Phase II trial completed	(158)
TRACP 5b	Bone resorption marker	Reflects osteoclast activity	Vitamin B1 28 mg/day for 1 month	Pilot single-arm trial	(159)
β-CTX, P1NP	Bone turnover markers	β-CTX for resorption, P1NP for formation; used to monitor intervention response	Moderate static magnetic field (MMF) exposure	RCT completed (ChiCTR2100048604)	(160)
IL-1β, IL-6, TNF-α	Inflammatory cytokines	Promote osteoclastogenesis and bone resorption	Drynaria fortunei extract via NLRP3 inflammasome suppression	Preclinical/observational	(22)
NLRP3 inflammasome	Inflammatory pathway	Activates caspase-1 and IL-1β; central in inflammatory bone loss	Regulated via SIRT1/Notch1 signaling	Mechanistic/preclinical	(22)
18F-NaF PET SUVmean	Imaging biomarker	Reflects vertebral bone turnover activity; high values suggest metabolic dysfunction	Colchicine 0.5 mg/day for 3 months	2×2 factorial RCT	(161)

Summary of key inflammation and oxidative stress–related biomarkers and corresponding therapeutic strategies evaluated in clinical trials for osteoporosis. MDA, malondialdehyde; 8-OHdG, 8-hydroxy-2’-deoxyguanosine; RANKL, receptor activator of nuclear factor kappa-B ligand; OPG, osteoprotegerin; etc.

Malondialdehyde (MDA) and 8-hydroxy-2′-deoxyguanosine (8-OHdG) are well-established markers of lipid and DNA oxidative damage, respectively. Elevated levels of MDA have been observed in iron-overloaded thalassemia patients with low bone mineral density (BMD), and supplementation with melatonin (20 mg/day for 12 months) significantly reduced MDA levels while improving lumbar spine BMD (157). Similarly, tocotrienol—a vitamin E isoform with potent antioxidant properties—has been shown to decrease urinary 8-OHdG and the serum sRANKL/OPG ratio, both of which are indicative of oxidative-induced bone resorption (158).

Among bone turnover markers, Tartrate-resistant acid phosphatase 5b (TRACP 5b) and C-terminal telopeptide of type I collagen (β-CTX) reflect osteoclast activity, whereas P1NP reflects bone formation. In a pilot study, vitamin B1 supplementation significantly reduced TRACP 5b levels in middle-aged adults (159). Additionally, a randomized controlled trial using moderate static magnetic field (MMF) exposure demonstrated decreased β-CTX and increased P1NP, suggesting a regulatory effect on bone remodeling (160).

Inflammatory cytokines such as IL-1β, IL-6, and TNF-α have also been implicated in osteoclast activation and inflammatory bone loss. An herbal intervention using Drynaria fortunei was found to suppress the NLRP3 inflammasome and downstream cytokines via the Sirtuin 1 (SIRT1)/Notch1 signaling pathway, indicating a potential anti-inflammatory bone-protective mechanism (22).

Notably, 18F-sodium fluoride positron emission tomography (18F-NaF PET) imaging offers a non-invasive method for assessing vertebral bone turnover. In a factorial clinical trial, patients with type 2 diabetes mellitus receiving colchicine (0.5 mg/day) exhibited a significant reduction in PET SUVmean values, highlighting the anti-inflammatory modulation of bone metabolism (161).

Incorporation of oxidative stress and inflammation-related biomarkers into clinical frameworks may enhance the precision of osteoporosis diagnosis and therapeutic monitoring. Moreover, emerging evidence supports the potential of adjunctive interventions—such as melatonin, tocotrienol, colchicine, and magnetic field therapy—as complementary strategies to conventional antiresorptive and anabolic treatments.

### 2.6 microRNAs, epigenetics, and mitochondria: emerging modulators of bone remodeling

Recent evidence highlights the crucial role of emerging molecular regulators—particularly non-coding RNAs, epigenetic modifications, mitochondrial dynamics, and extracellular vesicle (EV) signaling—in the pathogenesis of osteoporosis (162).

Among non-coding RNAs, several miRNAs modulate osteoblast and osteoclast differentiation through key signaling pathways. miR-96 promotes osteogenesis via activation of Wnt/β-catenin signaling, whereas miR-125b impairs bone formation by inhibiting BMP signaling (163, 164). miR-214, enriched in osteoclast-derived EVs, suppresses osteoblast activity by targeting activating transcription factor 4 (ATF4), linking miRNA signaling to cross-lineage regulation during bone remodeling (165). LncRNAs can influence osteogenesis by modulating genetic components involved in bone metabolism. Metastasis-associated lung adenocarcinoma transcript-1 (MALAT-1) protects against osteoporosis by inhibiting osteoclast differentiation via TEA domain family member 3 (Tead3)/NFATc1 signaling (166). Differentiation Antagonizing Non-Protein Coding RNA (DANCR) promotes osteoporosis by suppressing osteogenic differentiation through the inhibition of catenin beta 1 (CTNNB1) and the Wnt/β-catenin pathway (167). Additionally, circulating SNHG1 is significantly downregulated in postmenopausal osteoporosis and may serve as a diagnostic and prognostic biomarker (168).

Epigenetic regulation contributes to OP through dynamic DNA and histone modifications. For instance, hypermethylation of the BMP2 promoter suppresses osteogenic gene expression, and the histone methyltransferase enhancer of zeste homolog 2 (EZH2) represses RUNX2 through histone H3 lysine 27 trimethylation (H3K27me3) deposition (169, p. 3). In contrast, SIRT1 promotes OB function by enhancing Wnt signaling and mitigating oxidative stress (170).

Mitochondrial dysfunction in bone cells is increasingly recognized as a driver of skeletal aging and OP. Loss of PTEN-induced kinase 1 (PINK1) impairs mitophagy and osteogenesis, while Sirtuin 3 (SIRT3) preserves mitochondrial integrity and protects against bone loss (171, 172). Resveratrol has shown therapeutic promise by improving OB mitochondrial function via PI3K/AKT signaling.

Extracellular vesicles play a pivotal role in the pathogenesis and diagnosis of osteoporosis. EVs derived from BMSCs regulate bone homeostasis by delivering bioactive molecules such as miR-328-3p and ring finger protein 146 (RNF146), which activate the Wnt/β-catenin pathway via Axin1 inhibition (173). Additionally, elevated levels of miR-31a-5p in aged BMSC-derived EVs impair osteogenesis and promote adipogenesis, contributing to age-related bone loss. Circulating EV-associated miRNAs, including t miR-3-766p and miR-3-1247p, have emerged as potential diagnostic biomarkers for osteoporosis (174). These integrated findings provide a comprehensive framework for understanding bone homeostasis and suggest multiple actionable targets for the development of more effective anti-osteoporotic strategies.

### 2.7 Critical issues and future challenges

While this review synthesizes the mechanistic and therapeutic landscape of inflammation and oxidative stress in osteoporosis, several unresolved controversies and limitations must be acknowledged. These challenges highlight key gaps in our current understanding and point to important directions for future research.

The clinical application of anti-inflammatory biologics for osteoporosis treatment faces significant variability in patient responses. TNF-α inhibitors like infliximab show inconsistent effects on bone mineral density across different studies (88, 93), while IL-6 blockers such as tocilizumab demonstrate mixed impacts on bone turnover markers (102). This heterogeneity likely stems from differences in patient characteristics, disease subtypes, and underlying molecular mechanisms that remain poorly characterized. The development of predictive biomarkers to identify patient subgroups that would benefit most from these therapies represents a crucial unmet need.

The dual roles of inflammatory cytokines in bone metabolism present both opportunities and challenges for therapeutic intervention. IL-17, for instance, promotes osteoclastogenesis through RANKL upregulation (19) while simultaneously enhancing osteoblast activity via JAK2/STAT3 signaling (85). Similarly, IL-6 exhibits context-dependent effects, suppressing osteoblast differentiation *in vitro* (79) while supporting bone formation *in vivo* through gp130-mediated pathways (16). These paradoxical effects underscore the need for more nuanced therapeutic strategies that can selectively target detrimental inflammatory signaling while preserving beneficial aspects of cytokine activity.

Despite promising preclinical results, translating antioxidant therapies to clinical practice faces multiple hurdles. Natural compounds like resveratrol and icariin suffer from poor bioavailability and inconsistent efficacy in human trials (23). Synthetic antioxidants such as ebselen and N-acetylcysteine, while effective in reducing oxidative stress in experimental models (21, 24), lack comprehensive long-term safety data in clinical populations. Furthermore, the potential for dose-dependent effects—as seen with vitamin E where high doses may paradoxically impair bone formation (146, 148) - necessitates careful optimization of treatment regimens.

Several fundamental questions about the interplay between inflammation, oxidative stress, and bone remodeling remain unanswered. The precise role of mitochondrial dysfunction in osteoporosis pathogenesis, while increasingly recognized (44), requires further elucidation, particularly regarding potential therapeutic strategies to restore mitochondrial homeostasis. Similarly, the complex epigenetic regulation of bone metabolism through mechanisms such as DNA methylation and non-coding RNAs (165, 169) presents both challenges and opportunities for targeted interventions. Emerging areas like the gut-bone axis (20) offer exciting possibilities but demand more rigorous clinical validation.

Future research should focus on precisely revealing the dynamic changes of inflammation and oxidative stress at different stages of osteoporosis, identifying specific biomarkers, and developing new drugs that can selectively regulate these pathways. Furthermore, multidimensional intervention strategies, such as combining anti-inflammatory drugs with antioxidants, targeting mitochondrial function restoration, stem cell transplantation, and tissue engineering technologies, may further enhance treatment outcomes. By integrating genomics, proteomics, and metabolomics, a precision diagnosis and treatment system for osteoporosis can be established, significantly improving early diagnosis and personalized treatment capabilities. At the same time, in-depth evaluation of the long-term safety and efficacy of different interventions, coupled with close collaboration between basic research and clinical practice, will lay a stronger theoretical and practical foundation for osteoporosis prevention and treatment, ultimately shifting from passive treatment to active prevention.
